# Revisiting human T-cell lymphotropic virus types 1 and 2 infections among rural population in Gabon, central Africa thirty years after the first analysis

**DOI:** 10.1371/journal.pntd.0006833

**Published:** 2018-10-25

**Authors:** Melanie Caron, Guillaume Besson, Cindy Padilla, Maria Makuwa, Dieudonne Nkoghe, Eric Leroy, Mirdad Kazanji

**Affiliations:** 1 Centre International de Recherches Medicales de Franceville (CIRMF), Franceville, Gabon; 2 Ministere de la Sante, Libreville, Gabon; 3 Institut Pasteur de la Guyane, Reseau International des Instituts Pasteur, Cayenne, French Guiana; Hospital Universitário Professor Edgard Santos, BRAZIL

## Abstract

HTLV-1 infection is considered as highly endemic in central Africa. Thirty years ago, a first epidemiological study was performed in Gabon, central Africa, and revealed that the prevalence varied from 5.0 to 10.5%. To evaluate current distribution of HTLVs in Gabon, 4.381 samples were collected from rural population living in 220 villages distributed within the 9 provinces of country. HTLVs prevalence was determined using two ELISA tests and positive results were confirmed by Western Blot. The overall HTLV-1 seroprevalence was of 7.3% among the rural Gabonese population; with 5.4% for men and 9.0% for women. Prevalence of HTLV-1 differed by province, ranging from 2.3% to 12.5% into the rain forest. Being a woman older than 51 years represented a high risk for HTLV-1 acquisition. Hospitalization, operation/surgery, transfusion and medical abortion or fever, arthritis and abdominal pain are also significant risk factors. In addition, 0.1% of samples were found as HTLV-2 positive, while 12.0% had an indeterminate HTLV serological pattern. HTLV-3 and HTLV-4 were not found. Phylogenetic analysis was performed on 87 samples and demonstrated that HTLV-1 present in Gabon belongs mostly to subtype B, however the rare subtype D was also found. Altogether, our results demonstrate that almost thirty years after the first epidemiological study prevention of HTLVs infection is still an issue in Gabon.

## Author summary

HTLV-1 is endemic in Africa where it infects millions of individuals and causes Adult T Leukemia. Thirty years ago, an epidemiological study was performed in Gabon, where HTLV-1 is endemic. This revealed a high prevalence of this retroviral infection. Thirty years later, we performed a larger study in the general Gabonese population. This reveals that this oncogenic retrovirus is still highly frequent in this country and that prevalence remains unchanged. It would be extremely important to inform the general population about this infection in order to be able to decrease this retrovirus transmission.

## Introduction

Human T-cell leukemia virus type 1 (HTLV-1) and -2 (HTLV-2) belong to the Primate T Lymphotropic Virus group (PTLV), sharing common epidemiological and biological properties, and in particular T-lymphocytes tropism [1]. Isolated in 1980, HTLV-1 is the causative agent of adult T-cell leukemia/lymphoma (ATL) [2] and tropical spastic paraparesis/HTLV-1-associated myelopathy (TSP/HAM) [3]. It has also been associated with a variety of inflammatory diseases, including pediatric infectious dermatitis [4], uveitis [5] and some cases of arthropathy [6] or polymyositis [7]. Two years after, HTLV-2 was discovered and is potentially responsible for rare neurological syndromes that are clinically related to TSP/HAM [8–9]. Recently, two new retroviruses, i.e. HTLV-3 and HTLV-4, were identified in Cameroon. However, no disease has yet been associated to these viral infections [10–11].

HTLV-1 is not ubiquitous but endemic in some areas and among some ethnic groups. HTLV-1 is highly endemic, in southern Japan, sub-Saharan Africa and the Caribbean Basin as well as parts of South America or Middle East [12]. The global estimation reaches 5 to 10 million of infected persons worldwide, and 2–8% will develop a severe HTLV-1-associated disease in their lifetime [13–14]. Meanwhile, HTLV-2 has been recognized to be endemic among various American Indian populations [15–16], and likewise for the past 15–25 years, among the intravenous drug users in Europe and North America [17–18]. Furthermore, sporadic cases of HTLV-2 infection have been detected in west and central Africa among isolated rural populations as Pygmies, suggesting an ancient presence of HTLV-2 in central Africa [19–21].

HTLV-1 and HTLV-2 infection is spread through sexual transmission mainly, from man to woman; parenteral transmission as by needle sharing, blood transfusion or organ transplants and mother-to-child transmission associated with prolonged breast-feeding [22–25]. Diseases associated with HTLV-1 have been reported after blood transfusion and develop rapidly after seroconversion [26]. Indeed, TSP/HAM post-transfusion cases appear to be more severe and to evolve faster than non-post-transfusion cases [27].

Mother-to-child transmission has been reported as predominant route in endemic areas. HTLV-1 seroprevalence increase with the age, especially among women [28]. In Gabon, a central African country, considered as endemic area for HTLV-1 infection, seroprevalence varies considerably between sex, age and region [29]. The first epidemiological survey regarding HTLV-1 prevalence in Gabon and more specifically in Franceville, Eastern Gabon, was carried in 1986. 1874 individuals were analyzed including adult and children from urban and rural areas. Results showed that the prevalence varied from 5.0% to 10.5% [30]. Ten years ago, we reported a global prevalence of HTLV-1 among pregnant women of 2.1%, reaching up to 5.0% in some areas of this country [31]. Moreover, mother-to-child transmission and blood transfusion appeared to be the main routes of HTLV-1 infection among hospitalized children [32–34] and interfamilial transmission was also reported [35].

To determine the situation and evolution of HTLV-1 infection in Gabon thirty years after the first epidemiological survey, we performed a new epidemiological study and determined the prevalence of HTLVs throughout the country among rural adult population. HTLV-1 proviral load, a major factor for the viral transmission and disease evolution [36], was also measured in infected individuals. Molecular epidemiology using LTR sequence analysis from 87 strains demonstrates that virus from this country belongs to different subtypes including the rare HTLV-1 subtype D. More importantly, we demonstrate that HTLV seroprevalence is similar to that shown almost 30 years ago, thus demonstrating that HTLV infection is still an issue in Gabon.

## Methods

### Ethics statement

The study protocol was reviewed and approved by the Gabonese Ministry of health (Research authorization n°00093/MSP/SG/SGAQM). The Health Director and the Governor of each province received written information from the traditional chief of each village. Likewise, future research studies were orally described to all participants and an individual written consent was signed before blood sampling. Parents’ written consent was obtained for the participating children.

### Study area and population

Gabon is localized in central Africa, crossed by the equator and covered by nearly 80% of rain forest. This country has a surface area of 267.667 km^2^ for about 1.5 million of inhabitants (5.6 inhabitants/km^2^), including 73% living in urban areas. Gabon is divided into 9 provinces for a total of 2.048 villages. Villages are predominately located along roads and rivers. Few have more than 300 inhabitants. The main population activities are subsistence farming, hunting, gathering and fishing. Geographically, the country is constituted of four ecosystems: the Forest composed of deep forest generally characterized by presence of “Okoumé” trees (*i*.*e*. Mountain Forest, Interior Forest and North-East Forest nearby the borders of Cameroon and Republic of Congo); the Grassland with mountains and plateaus (*i*.*e*. between Coastline and Mountain Forest); the dry Savannah (*i*.*e*. in South-East, South-West and Interior of Gabon) and the wide “Lakeland”, a coastal and continental marine ecosystem (*i*.*e*. in West of Gabon).

The survey covered 220 randomly selected villages (range from 10 to 41 villages per province) located in the different ecosystems. It focused on rural villages with fewer than 300 inhabitants located in the 9 administrative regions of Gabon. Besides, all the villages were geo-located. A multidisciplinary team including a doctor from the Gabonese Ministry of Health, a nurse, an epidemiologist, a virologist and laboratory technicians took place during nine field missions between June 2005 to September 2008. Healthy volunteers older than 15 years and who had been living in the village for more than one year were eligible for the study.

### Epidemiological and medical investigations

Each volunteer participant answered to an anonymous questionnaire for subsequent epidemiological investigation on their geographical location, sociodemographic characteristics or lifestyle. Participants were also interviewed on their history of medical past. Only age and gender were collected for children. In addition, a free medical examination and basic medicine including clinical neurological examination were provided to all participants and non-participants. Blood smears for malaria diagnosis and field blood typing were also proposed.

### Blood collection

Blood samples were usually collected in the villages’ local healthcare centers. They were stored into 7 mL Vacutainer tubes containing EDTA (VWR International, France). Tubes were transported to the field laboratory for centrifugation (10 min, 2 000*g*) on a daily basis. Plasma and Buffy-coat were stored at -20°C until the end of field mission then, transferred on dry ice to the “Centre International de Recherches Medicales de Franceville” (CIRMF) and kept at -80°C until analysis.

### Determination of HTLV serological status

Two enzyme-linked immunosorbent assays (Platelia, HTLV-I New, Bio-Rad, France; Vironostika, HTLV-1/2, Bio-Merieux, France) were first used for HTLV serology. HTLVs positive or borderline-positive samples were tested by western blotting (HTLV Blot 2.4, MP Diagnostics Suisse S.A., Switzerland) to differentiate HTLV-1 and HTLV-2 status from HTLV indeterminate serological status.

### HTLV-1 proviral load

DNA was extracted from 200μl of Buffy-coat with the QIAamp DNA mini kit (Qiagen, Courtaboeuf, France) according to the manufacturer’s recommendations. HTLV-1/2 proviral load was measured by a multiplex real-time PCR assay involving a molecular beacon probe for simultaneous detection, differentiation, and quantification of HTLV-1, -2, and -3 and STLV-1 and -3, as previously described [37]. Samples with indeterminate HTLV western blot profile were also tested using the same multiplex real-time PCR assay.

### Molecular and phylogenic analysis

Polymerase chain reaction (PCR) amplification was performed with HTLV-1 or HTLV-2 specific primers located within the long terminal repeat (LTR) or the envelope glycoprotein (*env*) sequence, as previously described [38–39]. PCR products were directly sequenced.

For phylogenic HTLV-1 analysis, LTR sequences were analyzed and aligned with MEGA package, the editor program [40]. Trees were inferred by the Bayesian method implemented in MrBayes version 3.1 software with the Jones, Taylor and Thornton model and the rtREV model of evolution and gamma distributed rates at sites, with one million generations and burn-in of 2.5%. Bayesian parameters were examined with the Tracer program (http://tree.bio.ed.ac.uk/software/tracer/), and all estimated sample sizes were greater than 545 [41]. Phylogenic HTLV-2 analysis of the *env* sequences (accession numbers JF270595 to JF270597) confirmed the results obtained with the LTR sequences (accession numbers JF270592 to JF270594).

### Nucleotide sequence accession numbers

Nucleotide sequences obtained from the LTR and *env* gene of HTLV-1/-2 were assigned GenBank accession numbers JF270481 to JF270597.

### Statistical methods

To describe our rural Gabonese population, we assessed crude HTLVs seroprevalence. In order to reflect the linear association between HTLV-1 infection (*i*.*e*. prevalence and proviral load) and the sociodemographic characteristics (*i*.*e*. province, age and sex), a trend test across ordered groups was used. HTLV-2 statistical analysis could not be performed because of a low number of infected samples (n = 5). All statistical analyses were performed using STATA software version 10 (STATA Corporation, College Station, Texas, USA). Statistically significance was assumed at p≤0.05.

For the explanatory univariate analysis, we examined potential associations between HTLV-1 seroprevalence (*i*.*e*. HTLV-1 *vs*. negative status or HTLV-1 *vs*. indeterminate serological status) and risk factors (*i*.*e*. sociodemographic characteristics, lifestyle or medical past) using Chi-square test (χ^2^) or Fisher’s exact test when appropriate. We performed two separate binary logistic regression models HTLV-1 *vs*. negative status or HTLV-1 *vs*. indeterminate status as outcome. In univariate analysis, strength association between serological status and risk factors was estimated as odds ratio (OR) with 95% confidence interval (95%CI).

For explanatory multivariate analysis, a multinomial logistic regression model was built using all variables significantly associated with serological status in both previous explanatory univariate analysis. In this model, the referent group was HTLV-1 compared to negative status *vs*. HTLV-1 or indeterminate serological status *vs*. HTLV-1. Independent associations were also investigated and selected for input into a multivariable logistic regression analysis while controlling for confounders in the model at a probability threshold of ≤0.10 level. In multivariate analysis, strength associations between serological status and risk factors were estimated as multinomial with 95% confidence intervals (95%CI).

## Results

### Survey participation and study population

**Fig 1:**
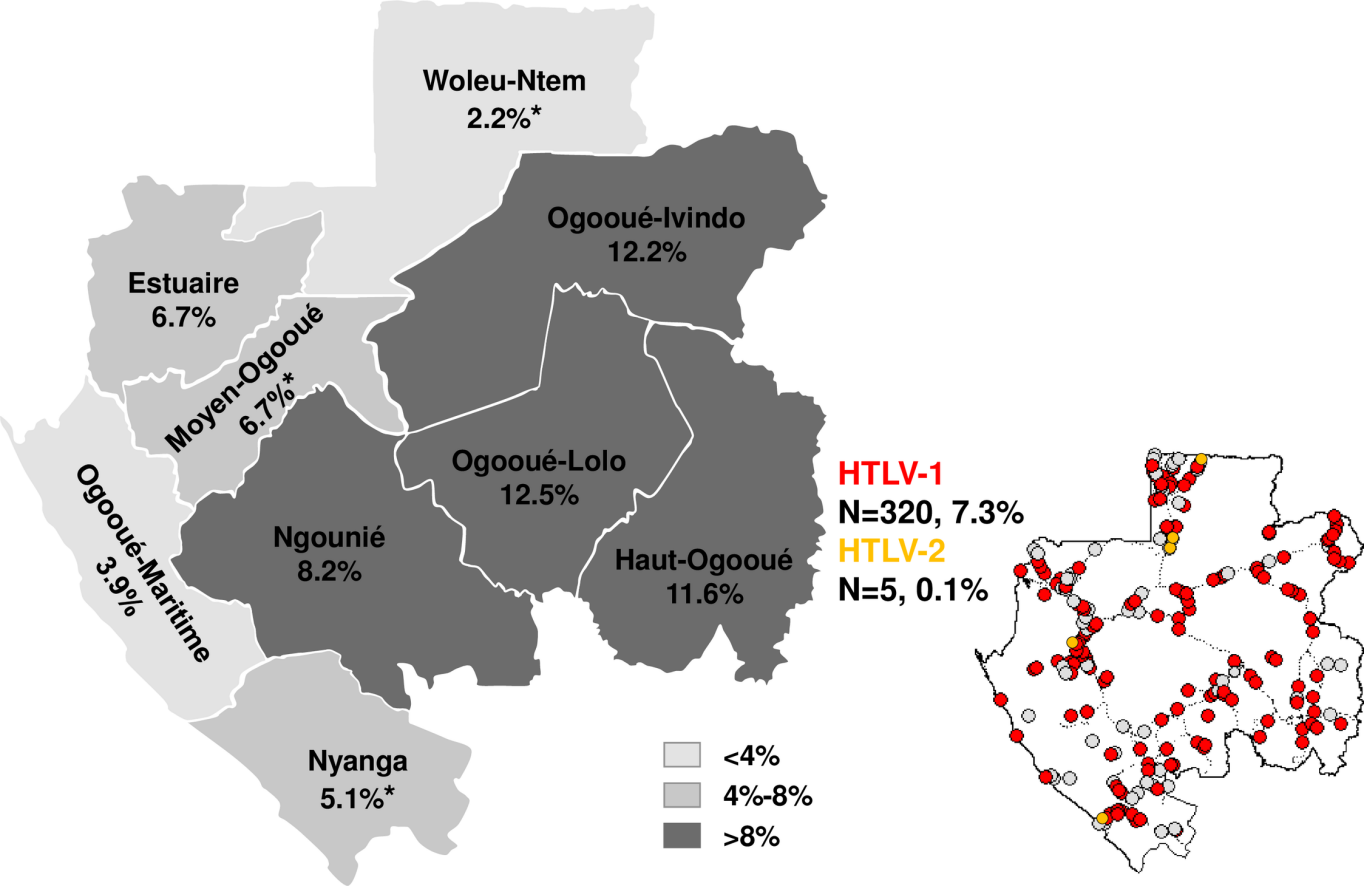
Spatial distribution of HTLVs infection and seroprevalence among rural population in Gabon, central Africa. *: Province with HTLV-2 infection; N: Number of HTLV-positive sample. Dotted line: main roads. Red circle: villages with HTLV-1 infection. Orange circle: villages with HTLV-2 infection. White circle: village with no HTLV positive sample. Location of villages was strictly geo-referenced and maps were generated by MAPINFO software (https://www.pitneybowes.com/us/location-intelligence/geographic-information-systems/mapinfo-pro.html).

4,381 participants were enrolled throughout the whole country, representing 1.1% of the rural Gabonese population (n = 387 670 inhabitants). Blood samples and sociodemographic data were collected for all participants. Male/Female sex ratio was of 0.90 and mean age was 47±14 years old (range from 15 to 90 years old). There was no mean age significant difference in the different location. Participants were living in Lakeland (n = 447, 10.2%), Savannah (n = 451, 10.3%), Grassland (n = 924, 21.1%), and Forest (n = 2 559, 58.4%), subdividing into Mountain Forest (n = 403, 9.2%); Interior Forest (n = 1 323, 30.2%) and North-East Forest (n = 833, 19.0%). The investigated villages were randomly selected, covering 10.7% of all villages in Gabon (Fig 1, right).

### HTLV spatial distribution, seroprevalence and HTLV proviral load

HTLV-1 and HTLV-2 prevalence was 7.3% (n = 320) and 0.1% (n = 5), respectively (Fig 1). HTLV-1 infection was distributed through all different Gabonese ecosystems (Fig 1, right panel) and classified into low (<4%), intermediate (4–8%) and high (>8%) prevalence (Fig 1, left part). In addition, HTLV-2 infections were observed in province of Moyen-Ogooué (n = 1), Nyanga (n = 1) and Woleu-Ntem (n = 3) (Fig 1, left part). Elevated HTLV-1 seroprevalence was observed within the Forest and dry Savannah in province of Ogooué-Ivindo, Ogooué-Lolo, Haut-Ogooué and Ngounié, intermediate within the Grassland and Savannah in Estuaire, Moyen-Ogooué and Nyanga. Lowest seroprevalence was found within the wide Lakeland in Ogooué-Maritime and the Forest in Woleu-Ntem.

Thus, overall HTLV-1 seroprevalence was 7.3% among rural Gabonese population; ranging from 2.3% to 12.5% by province (Table 1). HTLV-1 seroprevalence was significantly different between province as in Woleu-Ntem and Ogooué-Lolo (*p*<0.001). The odd ratio identified a strong risk for HTLV-1 acquisition when living in province of Haut-Ogooué (OR 1.74, 95%CI 1.23–2.45), Ogooué-Ivindo (OR 2.01, 95%CI 1.53–2.64) and Ogooué-Lolo (OR 1.98, 95%CI 1.45–2.71) compared with the other Gabonese provinces.

**Table 1:** Prevalence of HTLV-1 and indeterminate serological status by province among rural population in Gabon, central Africa.

Province	HTLV-1					IND				
	n/N	%	95% CI	OR	95% CI	n/N	%	95% CI	OR	95% CI
Estuaire	21/314	6.7	3.9–9.5	0.90	0.57–1.42	52/314	16.6	12.5–20.7	1.50	1.10–2.05
Haut-Ogooué	42/363	11.6	8.3–14.9	1.74	1.23–2.45	89/363	24.5	20.1–28.9	2.65	2.05–3.43
Moyen-Ogooué	46/684	6.7	4.8–8.6	0.90	0.65–1.24	38/684	5.6	3.9–7.3	0.39	0.28–0.55
Ngounié	30/365	8.2	5.4–11.0	1.15	0.78–1.70	53/365	14.5	10.9–18.1	1.15	0.85–1.56
Nyanga	22/429	5.1	3.0–7.2	0.66	0.42–1.03	33/429	7.7	5.2–10.2	0.58	0.40–0.84
Ogooué-Ivindo	76/622	12.2	9.6–14.8	2.01	1.53–2.64	81/622	13.0	10.4–15.6	1.11	0.86–1.43
Ogooué-Lolo	53/423	12.5*	9.3–15.7	1.98	1.45–2.71	38/423	9.0	6.3–11.7	0.70	0.50–0.99
Ogooué-Maritime	8/206	3.9	1.3–6.5	0.50	0.24–1.02	2/206	1.0	0.4–2.4	0.07	0.02–0.28
Woleu-Ntem	22/975	2.3*	1.4–3.2	0.24	0.15–0.37	141/975	14.5	12.3–16.7	1.32	1.07–1.62
**Total**	320/4381	7.3	6.5–8.1	-	-	527/4381	12.0	11.0–13.0	-	-

IND: HTLV indeterminate;

n: number of positive individuals;

N: number of tested individuals;

CI: confidence interval;

OR: odds ratio.

* Statistically significant difference (p<0,001), using Chi-square test (χ^2^) or Fisher’s exact test when appropriate.

A number of HTLV indeterminate western-blot results were observed (mean value 12.0%, ranging from 24.5% to 1.0%), including 8.3% of HTLV Gag-indeterminate pattern with reactivity to p24 and p19 proteins and a lack of reactivity against gp21 and gp46. The highest frequency of HTLV indeterminate serological status was found in Haut-Ogooué, province where elevated HTLV-1 seroprevalence was also observed (11.6%). The odd ratio was especially higher in this province (OR 2.65, 95%CI 2.05–3.43). The lowest HTLV indeterminate serological status was found in Ogooué-Maritime, province where HTLV-1 seroprevalence was also low (3.9%). HTLV-1 status was statistically higher (*p*<0.001) among women than among men (9.0% *vs*. 5.4%) (Table 2). HTLV-1 seroprevalence varies from 3.6% among the <25 years old to 9.6% among the >54 years old. Thus, being older than 54 years old and being a woman is a significant risk for HTLV-1 infection (OR 1.80, 95%CI 1.43–2.26).

**Table 2:** Prevalence of HTLV-1 by sex and age among rural population in Gabon, central Africa.

Age (years)	Male		Female		Total				
	n/N	% HTLV-1	n/N	% HTLV-1	n/N	% HTLV-1	95% CI	OR	95% CI
<25	4/144	2.8	10/249	4.0	14/393	3.6	1.8–5.4	0.45	0.26–0.78
25–34	11/285	3.9	15/319	4.7	26/604	4.3	2.7–5.9	0.54	0.36–0.81
35–44	10/392	2.6	34/414	8.2	44/806	5.5	3.9–7.1	0.70	0.50–0.97
45–54	23/393	5.9	49/535	9.2	72/928	7.8	6.1–9.5	1.10	0.84–1.45
>54	63/860	7.3	99/790	12.6	162/1650	9.8	8.4–11.2	1.80	1.43–2.26
Total	111/2074	5.4*	207/2307	9.0*	318/4381	7.3	6.5–8.1	-	-

n, number of positive; N, number of tested; CI, confidence interval; OR, odds ratio.

* Statistically significant difference (p<0,001), using Chi-square test (χ^2^) or Fisher’s exact test when appropriate.

A multiplex real-time PCR assay involving a molecular beacon probe for simultaneous detection, differentiation, and quantification of HTLV-1, -2, and -3 and STLV-1 and -3 was used on samples showing HTLV indeterminate WB profile. Neither HTLV-1, nor HTLV-2 or HTLV-3 could be found in any of these HTLV WB indeterminate samples.

**Fig 2:**
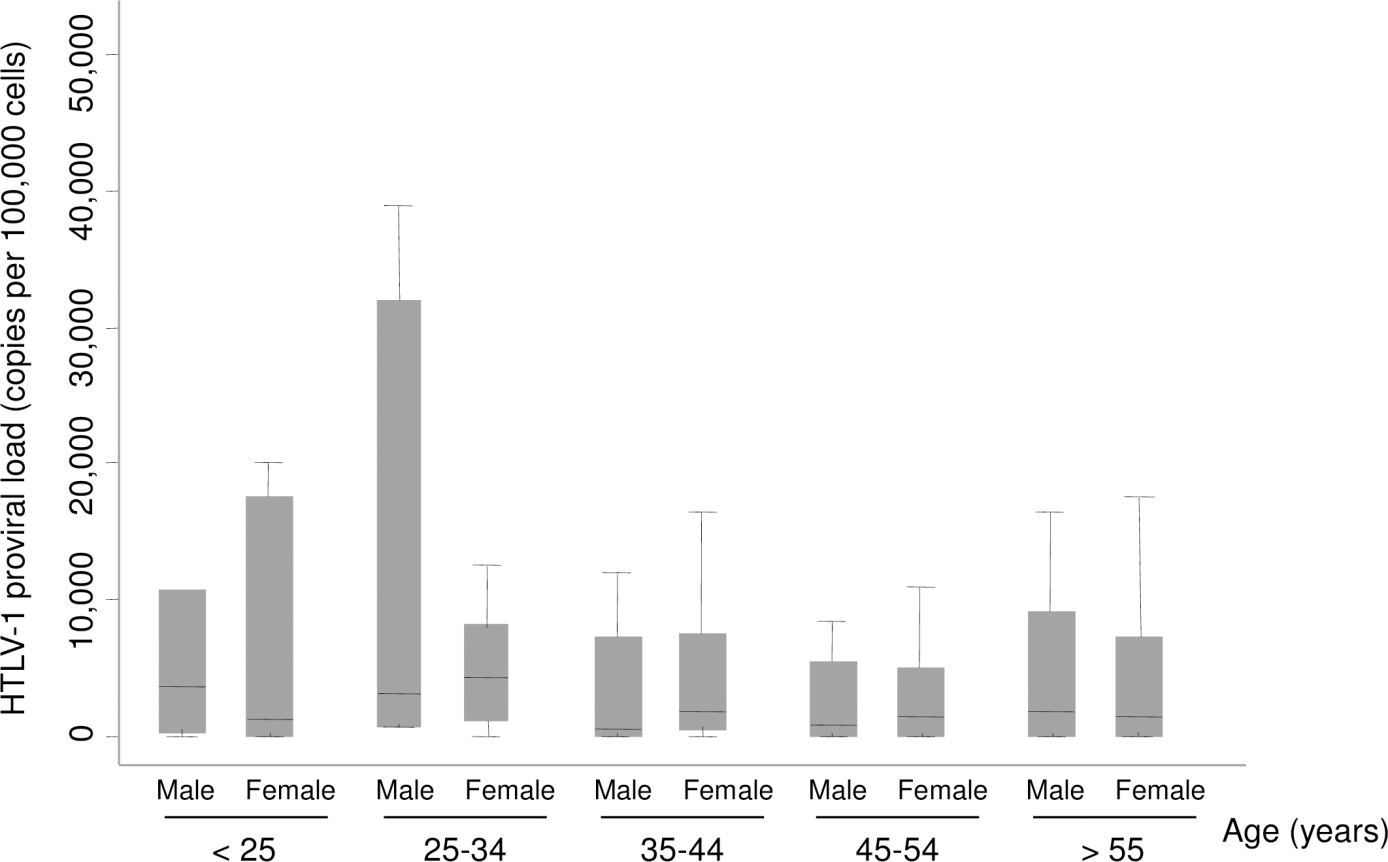
HTLV-1/2 proviral load by sex and age among rural population in Gabon, central Africa. Rectangle: Average magnitude of HTLV1/2 proviral load; Line: Median proviral load; Bar: Standard deviation. HTLV-1/2 proviral load was measured by a multiplex real-time PCR assay involving a molecular beacon probe for simultaneous detection, differentiation, and quantification of HTLV-1, -2, and -3 and STLV-1 and -3.

HTLV-1 proviral load was then measured using blood samples (Fig 2). Mean HTLV-1 proviral load was of 7.8x10^3^ ± 1.6x10^4^ copies per 10^6^ cells (range, from 1.1x10^1^ to 1.4x10^5^ copies per 10^6^ cells), with no statistical difference between province (*p* = 0.06). Although no significant correlation was found between proviral load and sex or age, it is still noteworthy that average magnitude of HTLV-1 proviral load was higher among the <35 years old (Fig 2).

### Epidemiological risk factors for infection with HTLV-1

To examine the influence of sociodemographic characteristics or medical past on HTLV-1 infection, we then performed analysis among the rural Gabonese population to compare HTLV-1 status with negative or HTLV indeterminate serological status (Table 3).

**Table 3:** Risk factors for infections with HTLV-1 compare to negative or indeterminate serological status among rural population in Gabon, central Africa.

Risk factors	Neg			HTLV-1	IND		
	*p value*	OR (95%CI)	No. (%)	No. (%)	No. (%)	OR (95%CI)	*p value*
Gender							
Male	**<0.0001**	1.00†	1692 (81.7)	111 (5.4)	268 (12.9)	1.00†	**<0.0001**
Female		1.73 [1.36–2.22]	1836 (79.7)	209 (9.1)	259 (11.2)	1.95 [1.45–2.62]	
Age							
≤51 years	**<0.0001**	1.00†	1992 (82.1)	130 (5.4)	303 (12.5)	1.00†	**<0.0001**
>51 years		1.89 [1.50–2.38]	1527 (78.7)	188 (9.7)	223 (11.5)	1.96 [1.48–2.59]	
Cook bushmeat							
No	0.39	1.00†	392 (92.5)	32 (7.6)	55 (63.2)	1.00†	0.98
Yes		1.18 [0.81–1.72]	2287 (91.2)	221 (8.8)	382 (63.4)	0.99 [0.61–1.64]	
Eat bushmeat							
No	0.62	1.00†	48 (94.1)	3 (5.9)	6 (66.7)	1.00†	1
Yes		1.52 [0.47–4.91]	2633 (91.3)	250 (8.7)	431 (63.3)	1.16 [0.27–4.97]	
Eat monkey							
No	0.19	1.00†	362 (89.8)	41 (10.2)	43 (51.2)	1.00†	**0.03**
Yes		0.79 [0.56–1.15]	3051 (91.8)	274 (8.2)	480 (63.7)	0.60 [0.37–0.97]	
Circumcision							
No	1	1.00†	8 (100)	0 (0)	0 (0)	1.00†	-
Yes		-	395 (94.9)	21 (5.1)	50 (70.4)	-	
Scarification							
No	0.11	1.00†	1669 (92.3)	139 (7.7)	227 (62.0)	1.00†	0.88
Yes		1.20 [0.95–1.53]	1773 (90.9)	178 (9.1)	297 (62.5)	0.98 [0.73–1.31]	
Tatoo							
No	0.83	1.00†	3185 (91.6)	292 (8.4)	485 (62.4)	1.00†	0.81
Yes		1.05 [0.65–1.62]	260 (91.2)	25 (8.8)	39 (60.9)	1.06 [0.60–1.85]	
Hospitalization							
No	**0.002**	1.00†	1379 (93.3)	99 (6.7)	213 (68.3)	1.00†	**0.006**
Yes		1.47 [1.14–1.90]	2060 (90.5)	217 (9.5)	309 (58.8)	1.51 [1.11–2.05]	
Operation, surgery							
No	**0.001**	1.00†	2523 (92.5)	204 (7.5)	382 (65.2)	1.00†	**0.009**
Yes		1.52 [1.18–1.94]	921 (89.1)	113 (10.9)	142 (55.7)	1.49 [1.09–2.03]	
Transfusion							
No	**0.002**	1.00†	3111 (92.1)	268 (7.9)	473 (63.8)	1.00†	**0.025**
Yes		1.67 [1.17–2.35]	319 (87.4)	46 (12.6)	50 (52.1)	1.62 [1.03–2.55]	
Medical abortion							
No	**0.009**	1.00†	994 (88.4)	130 (11.6)	158 (54.9)	1.00†	0.235
Yes		0.66 [0.47–0.91]	752 (92.0)	65 (8.0)	100 (60.6)	0.79 [0.52–1.19]	
Fever							
No	**<0.0001**	1.00†	1761 (93.5)	122 (6.5)	258 (67.9)	1.00†	**0.001**
Yes		1.86 [1.39–2.47]	777 (88.6)	100 (11.4)	120 (54.6)	1.76 [1.23–2.52]	
Arthritis							
No	**<0.0001**	1.00†	1758 (94.0)	112 (6.0)	244 (68.5)	1.00†	**<0.0001**
Yes		1.81 [1.41–2.34]	1556 (89.6)	180 (10.4)	222 (55.2)	1.76 [1.30–2.41]	
Abdominal pain							
No	**0.002**	1.00†	2400 (92.9)	182 (7.0)	342 (65.3)	1.00†	**0.002**
Yes		1.49 [1.14–1.93]	878 (89.9)	99 (10.1)	112 (53.1)	1.66 [1.18–2.33]	
Skin lesion							
No	**0.036**	1.00†	2395 (92.5)	195 (7.5)	317 (61.9)	1.00†	0.93
Yes		1.31 [1.00–1.70]	918 (90.4)	98 (9.7)	157 (61.6)	1.01 [0.74–1.40]	

Neg: HTLV Negative; IND: HTLV Indeterminate; OR, odds ratio; CI, confidential interval;

†, Referent.

Consuming non-human primate food was a risk factor associated with the indeterminate serological status compared with the HTLV-1 serological status (*p* = 0.03), while hunting and consuming bush meat were not statistically significant risks.

Hospitalization, surgery, transfusion and medical abortion represented risk factors for HTLV-1 acquisition (*p≤0*.*009*). Fever, arthritis, abdominal pain and skin lesion were significantly more frequent among HTLV-1 infected people (*p≤0*.*036*). Excepted medical abortion and skin lesion, risk factors observed among indeterminate persons were identical to those of HTLV-1 infected people (*p≤0*.*025*).

### Phylogenetic analyses

**Fig 3:**
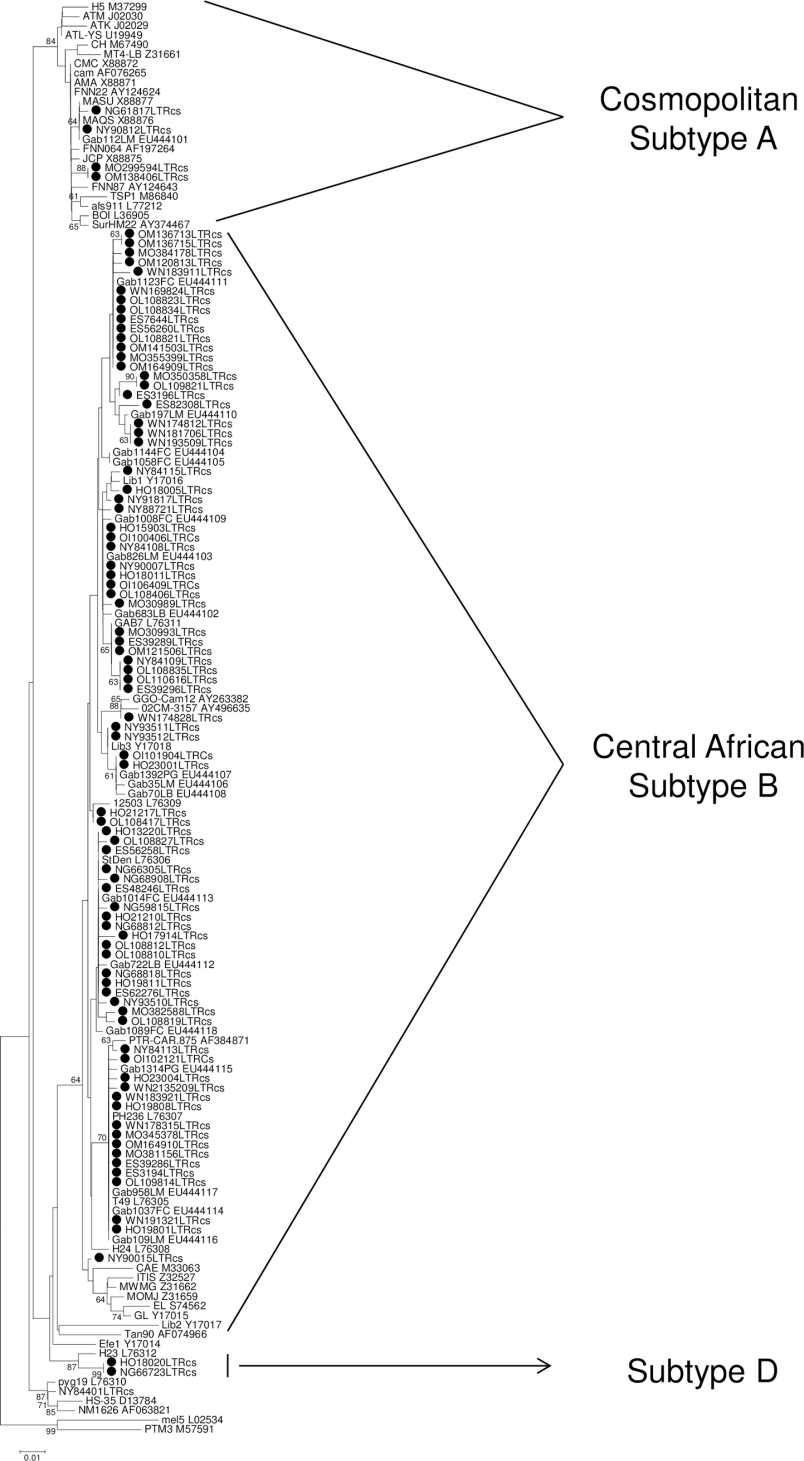
LTR phylogenetic analysis of HTLV-1 strains from Gabon. Phylogenetic tree was obtained on a 466bp LTR sequence using available sequences from Genbank. The 87 new HTLV-1 Gabonese strains are shown by bold circle. PTM3 (STLV-1 strain) was used to root the tree. The numbers along ancestral segments indicate the robustness of each node, as estimated by 1000 bootstrap samplings of the data. The Genbank accession numbers of the new sequences in the phylogenetic tree are JF270481 to JF270597.

Finally, phylogenetic analysis was conducted using a series of LTR sequences obtained from 87 samples (Fig 3). Consistent with Central Africa data, most strains belong to HTLV-1 subtype B, while some strains belong to subtype A (observed within Moyen-Ogooué, Ogooué-Maritime and Ngounié). Few other strains belong to the rare subtype D (observed within Ngounié and Haut-Ogooué provinces) previously found in Cameroon individuals and Gabonese non-human primates [42–43]. In addition, four samples belong to the HTLV-1 Cosmopolitan subtypes, present throughout the world.

## Discussion

Gabon is a country where HTLV-1 is endemic. Our study is the first where HTLV prevalence is determined throughout the country including all 9 administrative regions rather than only some representative regions or cities, as previously reported [30, 44]. HTLV-1 infection in Gabon was studied for the first time in 1986–87. At that time prevalence varied from 5.0% to 10.5% [30]. Our current results show that prevalence did not decrease and is almost higher than before. In another previous study performed among pregnant Gabonese women as well as in data reported 30 years ago, preventive measures, such as specific educational programs adapted to the local situation, aiming to decrease the spread and transmission of HTLV-1 in this area of high endemicity were proposed. Our present data demonstrate that prevention of HTLV-1 infection was not successful in this country.

Consistent with other reports, prevalence of HTLV-1 is significantly higher in women than in men (*p<0*.*001*). This finding is a well-known characteristic of HTLV-1 infection. Epidemiologic evidences suggest that sexual transmission of HTLV-1 is one of the predominant modes of transmission and occurred mainly from male to female. Sexual activity at an earlier age could be also a risk factor for HTLV-1 transmission. The highest prevalence in women might be due to an accumulation of sexual exposures with age [45–46].

We could not obtain blood from children and thus could not evaluate the prevalence of HTLV-1 in this population. However, 30 years ago, 684 children were included and a prevalence of 2% was found, with no difference between urban and rural areas [30]. Further epidemiological studies are urgently needed to evaluate and update the rate of HTLV-1 infection in children and to introduce preventive measures to decrease viral transmission.

Importantly, we show here that HTLV prevalence differs throughout the studied place and is much higher in forest areas. Similar data were obtained in our previous study performed in pregnant women from Gabon [31]. Furthermore, 30 years ago, age-adjusted prevalence rate of HTLV-1 was significantly higher in rural area than in urban one in Gabon [30]. Indeed, the high immigration from surrounding countries to Gabon as well as the rapid urbanization of several regions of the country could have led to socioeconomic and hygienic changes that might have modified the risk factors related to viral spread.

Furthermore, the reason for the high prevalence in some areas closed to other with low HTLV-1 prevalence is unknown. Same observation was found in the South of Japan where some regions with low HTLV-1 prevalence that can be very close to highly endemic region, especially in rural regions. As we indicated above, foci of high prevalence located near areas of low or very low endemicity is a hallmark of HTLV-1/-2 epidemiology but has never been explained. It could be related to genetic, environmental and socioeconomic factors (reviewed by [45–46]).

A previous report performed in Franceville described the fact that blood transfusion was a major risk for getting HTLV-1 in hospitals in Gabon [32]. It is unfortunate to observe that thirty years later, the same risk factor still exists (*p = 0*.*006*) throughout this country.

HTLV-1/2 infections with different HTLV subtypes, including rare ones [47] have been previously reported in Gabon [30, 31, 34, 41, 47–50], while STLV-1 strains from subtypes similar to human HTLV-1 were also described in this country [43, 51–54]. In the present study, HTLV-1 subtype D was also found in 2 separate regions of Gabon. These strains were obtained from 2 individuals living in Ngounié and Haut-Ogooué provinces where the zoonotic PTLV-1 transmission was previously described [55]. Few people have been found to be infected with this strain in central Africa [42]. In a previous study, we also reported the presence of this subtype in a girl who had been severely bitten by a *Cercopithecus nictitans* [55]. These data combined to the current study confirm that subtype D is still infrequent in central Africa, and probably related to a zoonotic transmission.

Recent reports have shown that, similar to foamy virus infection, being exposed to non-human primate and non-human primate food was a risk factor for HTLV-1 acquisition [55, 56]. Our results show that eating monkey food is a risk for PTLV-1 acquisition in Gabon (*p = 0*.*03*), although 5 times lower than being hospitalized. Medical examination and basic medicine including clinical neurological examination in the field were provided to all participants of the study. No clinical manifestation related to HTLV-1 such as clinical neurological dysfunction could be detected. However, there is an urgent need for a large survey and clinical investigations to evaluate exactly public health consequences of HTLV-1 infection in this highly endemic area of the world.

As previously reported with African sera/plasma samples, we observed a high number and various HTLV indeterminate patterns that were not HTLV-1/2 PCR positive [57–60]. Whether most of these samples are due to *P*. *falciparum* exposition as previously reported remains to be determined. Our multiplex real-time PCR primers allowed amplification of all PTLV-1/3 strains. Given that PTLV-4 is present in Gabon and because HTLV-4 carriers have an HTLV indeterminate western-blot pattern with very faint K55 and MTA-1 and GD21 envelope reactivity as well as a faint gag p24 reactivity [47], an alternative possibility could be that some of these individuals, living in the rain forest (Haut-Ogooué) are true HTLV-4 infected asymptomatic carriers.

In conclusion, our study demonstrates that HTLV-1 seroprevalence did not change 30 years after the first report, and that HTLV-1 subtype B is widely present in Gabon. We also showed that Gabon is one of the most highly endemic areas in the world for HTLV-I infection. Therefore, preventive measures to decrease spread and transmission of this human retrovirus are urgently warranted. These include: (1) systematic HTLV-I screening of blood donors (despite that data showing the high HTLV-1 prevalence in Gabon are available for 30 years, health authorities have not introduced yet a systematic HTLV-1 screening of the blood donors); (2) systematic screening of pregnant women in order to counsel them concerning the risk of HTLV-I transmission by prolonged breastfeeding (this information has already been transmitted to Gabonese ministry of health after our epidemiological study on pregnant women published in 2008); and (3) prevention of sexual transmission of HTLV-I by educational programs emphasizing the importance of using condoms to prevent all sexually transmitted diseases, including HIV infection, which is still spreading in this central African country.

Thus, it would be important to inform more efficiently the Gabonese population about this virus and its mode of transmission in order to allow a decreased transmission and a decreased prevalence in the future.

## Supporting information

S1 Checklist STROBE checklist. (DOC)
